# Analysis of detrended fluctuation function derived from continuous glucose monitoring may assist in distinguishing latent autoimmune diabetes in adults from T2DM

**DOI:** 10.3389/fendo.2022.948157

**Published:** 2022-09-20

**Authors:** Liyin Zhang, Qi Tian, Keyu Guo, Jieru Wu, Jianan Ye, Zhiyi Ding, Qin Zhou, Gan Huang, Xia Li, Zhiguang Zhou, Lin Yang

**Affiliations:** National Clinical Research Center for Metabolic Diseases, Key Laboratory of Diabetes Immunology, Ministry of Education, Department of Metabolism and Endocrinology, The Second Xiangya Hospital of Central South University, Changsha, China

**Keywords:** Latent autoimmune diabetes in adults, type 2 diabetes mellitus, beta-cell function, detrended fluctuation function, continuous glucose monitoring

## Abstract

**Background:**

We aimed to explore the performance of detrended fluctuation function (DFF) in distinguishing patients with latent autoimmune diabetes in adults (LADA) from type 2 diabetes mellitus (T2DM) with glucose data derived from continuous glucose monitoring.

**Methods:**

In total, 71 LADA and 152 T2DM patients were enrolled. Correlations between glucose parameters including time in range (TIR), mean glucose, standard deviation (SD), mean amplitude of glucose excursions (MAGE), coefficient of variation (CV), DFF and fasting and 2-hour postprandial C-peptide (FCP, 2hCP) were analyzed and compared. Receiver operating characteristics curve (ROC) analysis and 10-fold cross-validation were employed to explore and validate the performance of DFF in diabetes classification respectively.

**Results:**

Patients with LADA had a higher mean glucose, lower TIR, greater SD, MAGE and CV than those of T2DM (*P*<0.001). DFF achieved the strongest correlation with FCP (r = -0.705, *P*<0.001) as compared with TIR (r = 0.485, *P*<0.001), mean glucose (r = -0.337, *P*<0.001), SD (r = -0.645, *P*<0.001), MAGE (r = -0.663, *P*<0.001) and CV (r = -0.639, *P*<0.001). ROC analysis showed that DFF yielded the greatest area under the curve (AUC) of 0.862 (sensitivity: 71.2%, specificity: 84.9%) in differentiating LADA from T2DM as compared with TIR, mean glucose, SD, MAGE and CV (AUC: 0.722, 0.650, 0.800, 0.820 and 0.807, sensitivity: 71.8%, 47.9%, 63.6%, 72.7% and 78.8%, specificity: 67.8%, 83.6%, 80.9%, 80.3% and 72.4%, respectively). The kappa test indicated a good consistency between DFF and the actual diagnosis (kappa = 0.551, *P*<0.001). Ten-fold cross-validation showed a stable performance of DFF with a mean AUC of 0.863 (sensitivity: 78.8%, specificity: 77.8%) in 10 training sets and a mean AUC of 0.866 (sensitivity: 80.9%, specificity: 84.1%) in 10 test sets.

**Conclusions:**

A more violent glucose fluctuation pattern was marked in patients with LADA than T2DM. We first proposed the possible role of DFF in distinguishing patients with LADA from T2DM in our study population, which may assist in diabetes classification.

## Introduction

Latent autoimmune diabetes in adults (LADA), defined by the presence of islet autoantibody especially glutamic acid decarboxylase autoantibodies (GADA) and progressive islet function failure (1). LADA manifests a broad clinical phenotype between classic type 1 diabetes mellitus (T1DM) and classic type 2 diabetes mellitus (T2DM) (2). Consequently, a moderate proportion of LADA patients might be misdiagnosed as T2DM in the early stage. More importantly, the islet function in patients with LADA progresses much faster than that of T2DM (3). Once LADA patients develop insulin dependency, they will present much greater glucose fluctuation pattern than before. The standardized GADA testing is the recommended screening test for LADA because high GADA titer is correlated with accelerated decline of β-cell function (4). At present, early diagnosis of LADA patients remains a challenge since accurate and efficient islet antibody detection technology has not been widely carried out in many primary hospitals in China (5). The LADA international Expert Panel recommended that all newly diagnosed T2DM patients should be screened for GADA positivity and follow-up of progressing beta-cell failure annually, which might increase the burden of medical care (6). For this reason, there is increasing interest in exploring alternative approaches which may assist in LADA screening and diagnosis.

With gradual maturation of continuous glucose monitoring (CGM) technology and emerging clinical evidence in favor of CGM adoption (7), CGM ushered in a new era of glucose management. Currently, proposed CGM measures of interest such as standard deviation of glucose, mean amplitude of glucose excursions and time spent in given thresholds are mainly applied to reflect the instability of glucose and overall glycemic control. A previous study had reported higher glycemic variability metrics derived from CGM in patients with LADA than in T2DM (8). C-peptide (C-P), a reliable marker of β-cell function, may help discriminate diabetes types (9, 10). Moreover, C-P secretion is deemed to be associated with glycemic variability (11, 12). However, clinical detection of C-P is limited by the need to discontinue insulin. Although increasingly being used in clinical practice for the management of diabetes, few studies have investigated the role of CGM as a tool for the diagnosis of LADA, and whether the massive time series data provided by CGM can reliably distinguish LADA from T2DM is unknown. However, if use of CGM is found to reliably predict the diagnosis of LADA by GADA testing or C-P, then there would be multiple benefits to the patients (no admission, no time lost from work and no intravenous catheter or multiple blood draws) as well as economic advantages (costs of the admission, blood processing, C-P assays and personnel time), which represents an important step for CGM as an alternative and less burdensome approach for collaborative diagnosis of LADA.

Therefore, we aimed to:1) find a predictive indicator for serum C-P which can differentiate patients with LADA and T2DM through detrended fluctuation analysis, a modified random-walk analysis method using time series data derived from CGM (13); 2) evaluate its performance in identifying LADA from T2DM. Inspired by the usage of detrended fluctuation function (DFF) proposed by Liu et al. (14) in differentiating patients with T1DM and T2DM, we try to explore the same data-driven analysis in a more indistinguishable group consisting of LADA and T2DM. To our knowledge, studies regarding the glucose fluctuation of LADA are sparse, and this is the first study utilizing CGM metrics to differentiate LADA from T2DM.

## Materials and methods

### Study population

A total of 223 diabetes patients (71 with LADA and 152 with T2DM) from the outpatient department of the Second Xiangya Hospital, Central South University were included in this observational study. The inclusion criteria of patients with LADA were as follows: (1) diagnosis of diabetes according to the 1999 WHO criteria (15); (2) age > 18 years old; (3) insulin-independent for at least 6 months post-diagnosis; (4) GADA positivity; (5) no ketosis or ketoacidosis. The inclusion criteria of patients with T2DM were as follows: (1) diagnosis of diabetes according to the 1999 WHO criteria; (2) GADA negative; (3) age > 18 years old. Diabetes classification was made by a specialist and further confirmed by another one. Patients were excluded for one of following reasons: acute infection within 4 weeks prior to the recruitment, history of diabetic ketoacidosis in the past 3 months, abnormal liver/kidney function; with a comorbid autoimmune disease, pregnancy or preparing for pregnancy, receiving steroid therapy, and specific types of diabetes.

The study protocol was approved by the Ethics Review Committee of the Second Xiangya Hospital of Central South University (approval number: 2019-198; granted date: November 12, 2019), and it was carried out in accordance with the Declaration of Helsinki. Signed informed consent was obtained from each participant.

### Demographics and clinical measurements

Each patient underwent a physical examination that included measurements of height and weight, blood pressure. Demographics such as age, gender, duration of diabetes were collected. Blood samples for detecting lipid profiles (total cholesterol, high-density lipoprotein cholesterol, low-density lipoprotein cholesterol and total triglycerides), renal function (blood urea nitrogen, blood creatinine, uric acid), thyroid hormones (FT_3_, FT_4_, TSH), hemoglobin A1c (HbA1c), fasting blood glucose (FBG), fasting C-peptide (FCP) were drawn after 8-10h of fasting. A mixed-meal tolerance test (MMTT, 44.4% carbohydrates, 47.7% fat and 7.9% protein) was performed before 2-hour postprandial blood glucose (2hBG) and C-peptide (2hCP) measurements. For insulin treated patients, the long-acting insulin the night before the MMTT test was preserved and the morning prandial insulin was omitted. Patients treated with a pump continued their background basal rate but omitted the morning bolus. As for patients who were taking oral antihyperglycemic drugs (OADs) for glycemic control, they were required to discontinue insulin secretagogues (sulfonylureas or glinides) until the blood samples for detecting C-peptide and blood glucose levels were drawn.

Blood glucose levels, lipid profiles and other biochemical indicators were uniformly measured by an automatic biochemical analyzer. The level of HbA1c was determined by automated high-performance liquid chromatography (VARIANT II Hemoglobin Testing System; Bio-Rad Laboratories). Serum C-peptide levels were detected by a chemiluminescence method with an Adiva Centaur XP immunoassay system (Siemens, Germany).

### GADA assay

GADA was analyzed by a radioligand assay in our laboratory as previously described (4). As evaluated in the Islet Autoantibody Standardization Program (IASP 2012), the sensitivity and specificity of the assay were 78.0% and 96.7%, respectively.

### Continuous glucose monitoring

Dynamic glucose profiles were generated from the blinded CGM system (iPro2 with Enlite sensor, Medtronic MiniMed, Northridge, CA, USA). The glucose sensor of the CGM system (MMT-7008A) was inserted on the lateral upper arm and removed after 5-7 days, yielding a maximum daily record of 288 continuous sensor glucose values. With CGM, the participants were required to perform self-monitoring of blood glucose (SMBG) at least 4 times a day for calibration purposes. The CGM data were exported and analyzed using M-Smart software (CareLink iPro, Medtronic).

The time in range (TIR) was defined as the percentage of time spent in the normoglycemic range (3.9-10.0 mmol/L). Glycemic variability parameters included the standard deviation (SD), mean amplitude of glucose excursions (MAGE) and %CV (%CV= [(SD of glucose)/(mean glucose)]×100).

### Detrended fluctuation function

A DFF metric *F_d_(l)* was utilized, in which *l* was a segment size parameter used to adjust the performance of diabetes classification (14). A methodology of extended random-walk analysis known as detrended fluctuation analysis was adopted (13): first, (1) we integrated the glucose time series *x(t)*, the cumulative deviation was calculated, and *x(t)* was converted into a new series *y(t)*; Then (2) the new sequence *y(t)* was divided into m intervals (or windows) with equal length *n*, where *n* is the interval length, that is, the time scale; (3) used the least square method to linearly fit the local trend *y_n_(t)* for each sequence; (4) the local trend of each interval in *y(t)* was eliminated, the root mean square of the new sequence was calculated as *F(n)*; (5) changed the time scale *n* and repeated step 2,3 and 4. The calculation of *F(n)* was achieved by the MATLAB software.

### Statistical analysis

Normally distributed data were represented by mean ± SD, and skewed data after normality test (Shapiro-Wilk test) were represented by median and interquartile range (IQR). Independent sample *t* test or Mann-Whitney *U* test were used to compare differences between patients with LADA and T2DM. Spearman correlation analysis was employed to evaluate the correlation between the fluctuation function *F_d_(l)* and beta-cell function parameters including FCP and 2hCP. Moreover, classical CGM-derived glycemic parameters including TIR, mean glucose, SD, MAGE and CV were also evaluated and compared with *F_d_(l)*.

The receiver operating characteristics curve (ROC) analysis was performed to compare the classification performance of *F_d_(l)*, TIR, mean glucose, SD, MAGE and CV based on all study subjects. Ten-fold cross-validation was employed to test the stability of *F_d_(l)*. Moreover, ROC analysis was also performed in 116 insulin-treated patients (66 with LADA and 50 with T2DM). The kappa test was adopted to evaluate the classification consistency between actual classification and our study results.

A two-tailed test was performed, *P*<0.05 was considered statistically significant. SPSS 26.0 software (IBM corporation, Armonk, NY, USA) was used for statistical analysis. The computation of detrended fluctuation functions was performed in MATLAB 2020a (Mathworks, Inc., Natick, Massachusetts) for Windows.

## Results

### Demographics and clinical measurements

In total, 71 LADA and 152 T2DM patients were enrolled in the analysis, 61.0% of them were male. An average of 7-day CGM wearing was achieved, generating about 1,741 sensor glucose values per patient. The median age was 51.0 (42.0, 58.5) years. The average duration of diabetes was 5.0 (1.8, 9.0) years. Mean BMI level was 23.5 (20.9, 26.8) kg/m^2^. HbA1c was 8.2 (7.2, 9.8) % [66 (55, 84) mmol/mol], median FCP level was 1.22 (0.45, 2.13) ng/mL and 2hCP was 3.01 (1.14, 4.94) ng/mL. Glucose profiles derived from CGM were also listed in **Table 1**.

**Table 1:** Characteristics of all participants.

	LADA (*n* = 71)	T2DM (*n* = 152)	*P*
Sex (M/F)	35/36	101/51	0.015
Age (years)	48.0 (39.0, 57.0)	52.0 (43.5, 59.5)	0.239
Age of onset (years)	43.3 (33.5, 50.5)	45.0 (36.0, 53.0)	0.330
Duration (years)	4.3 (1.7, 10.0)	5.0 (2.0, 8.5)	0.921
BMI (kg/m^2^)	20.7 (19.3, 23.3)	25.1 (22.5, 27.3)	<0.001
SBP (mmHg)	113.0 (107.0, 129.0)	130.0 (120.0, 138.0)	<0.001
DBP (mmHg)	73.5 ± 10.5	84.0 ± 9.9	<0.001
FBG (mmol/L)	7.75 (6.00, 10.71)	6.52 (5.43, 8.37)	0.004
2hBG (mmol/L)	14.96 (10.82, 17.70)	10.77 (7.80, 13.28)	<0.001
HbA1c (%)	8.1 (7.3, 9.8)	8.3 (7.1, 9.9)	0.816
HbA1c(mmol/mol)	65 (56, 84)	67 (54, 85)	0.816
FCP (ng/mL)	0.31 (0.07, 0.48)	1.82 (1.20, 2.46)	<0.001
2hCP (ng/mL)	0.54 (0.09, 1.21)	4.18 (2.84, 6.24)	<0.001
Total cholesterol (mmol/L)	4.40 (3.68, 4.81)	4.89 (4.17, 5.38)	<0.001
Triglyceride (mmol/L)	0.77 (0.61, 1.15)	1.48 (1.07, 2.19)	<0.001
HDL-c (mmol/L)	1.38 (1.18, 1.63)	1.22 (1.03, 1.37)	<0.001
LDL-c (mmol/L)	2.60 (2.09, 2.98)	2.66 (2.21, 3.24)	0.142
BUN (mmol/L)	5.50 (4.70, 6.65)	5.00 (4.00, 6.15)	0.011
CR (umol/L)	68.0 (56.0, 75.0)	69.0 (57.0, 77.0)	0.469
UA (umol/L)	267.0 (216.7, 314.9)	327.5 (285.0, 412.5)	<0.001
Diabetes treatment, *n* (%)			
Diet/insulin sensitizers alone	2 (2.8)	54 (35.5)	–
DPP-4i/sulfonylureas	3 (4.2)	42 (27.6)	–
SGLT-2i	0	6 (3.9)	–
Insulin	66 (93.0)	50 (33.0)	–
CGM-derived metrics			
TIR (%)	62.3 (48.6, 79.4)	79.5 (65.7, 90.3)	<0.001
Mean glucose (mmol/L)	9.41 ± 2.13	8.31 ± 1.42	<0.001
SD (mmol/L)	3.31 (2.48, 3.99)	2.14 (1.62, 2.79)	<0.001
MAGE (mmol/L)	6.80 (5.60, 9.25)	4.60 (3.60, 5.60)	<0.001
CV (%)	34.8 (30.3, 39.1)	25.2 (20.9, 30.4)	<0.001
*F_d_(100)*	2.17 (1.67, 2.61)	1.36 (1.14, 1.69)	<0.001

Data are shown as mean ± SD, median (first quartile, third quartile) and ratio.

BMI, body mass index; SBP, systolic blood pressure; DBP, diastolic blood pressure; FBG, fasting blood glucose, 2hBG, 2-hour postprandial blood glucose; HbA1c, hemoglobin A1c; FCP, fasting C-peptide, 2hCP, 2-hour postprandial C-peptide; HDL-c, high-density lipoprotein cholesterol; LDL-c, low-density lipoprotein cholesterol; BUN, blood urea nitrogen; CR, blood creatinine; UA, uric acid; DPP-4I, dipeptidyl peptidase 4 inhibitors; SGLT-2i, sodium-dependent glucose transporter 2; TIR, time in range; SD, standard deviation of glucose; MAGE, mean amplitude of glucose excursions; CV, coefficient of variation.

### Optimal *l* selection and the relevant *F_d_(l)* values in LADA and T2DM patients

**Figure 1:**
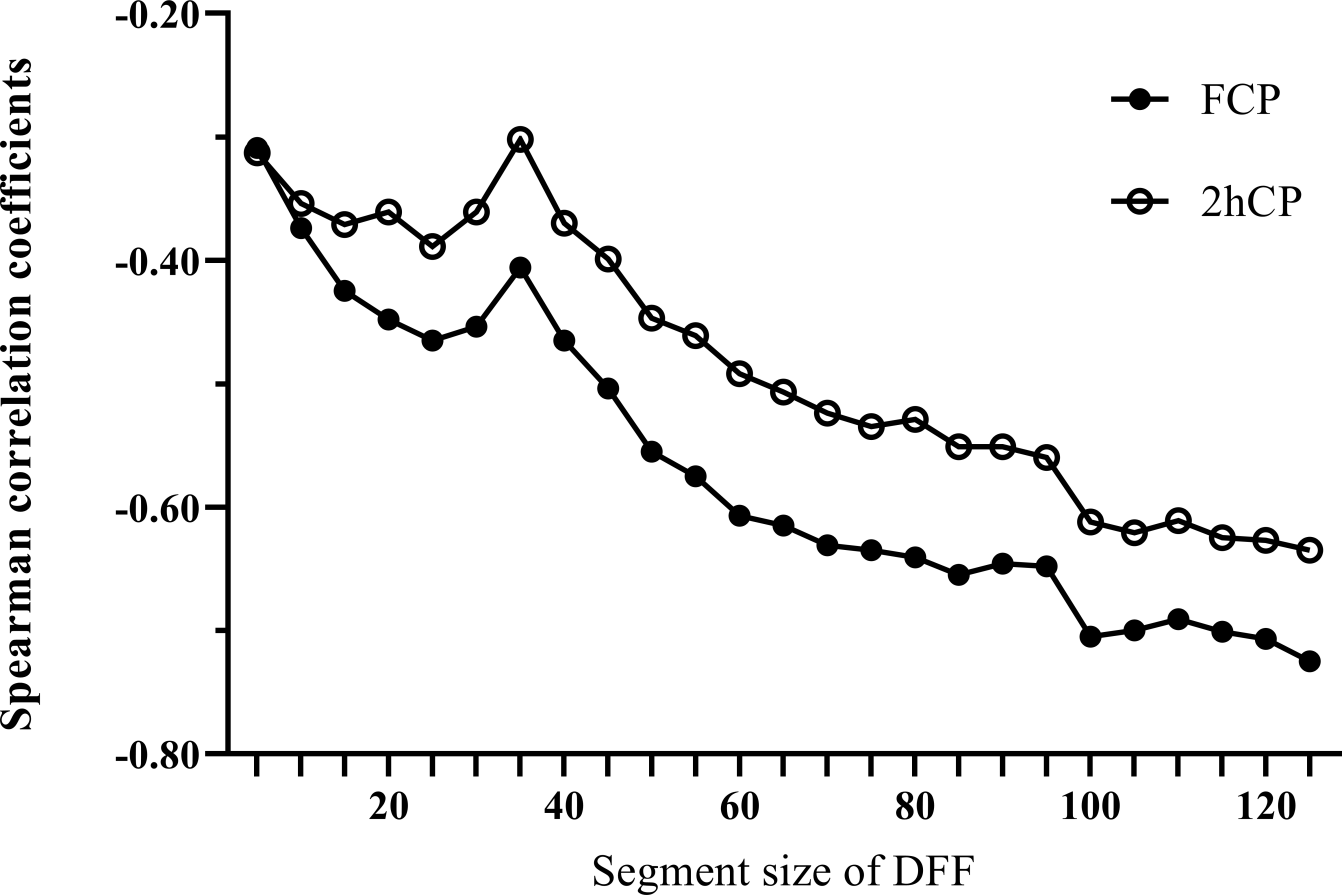
Spearman correlation curves between Fd(l) and beta-cell function indices (FCP and 2hCP). Black circles curve, correlation curve between FCP and Fd(l); white circles curve, correlation curve between 2hCP and Fd(l). FCP, fasting C-peptide; 2hCP, 2-hour postprandial C-peptide; DFF, detrended fluctuation function.

**Figure 2:**
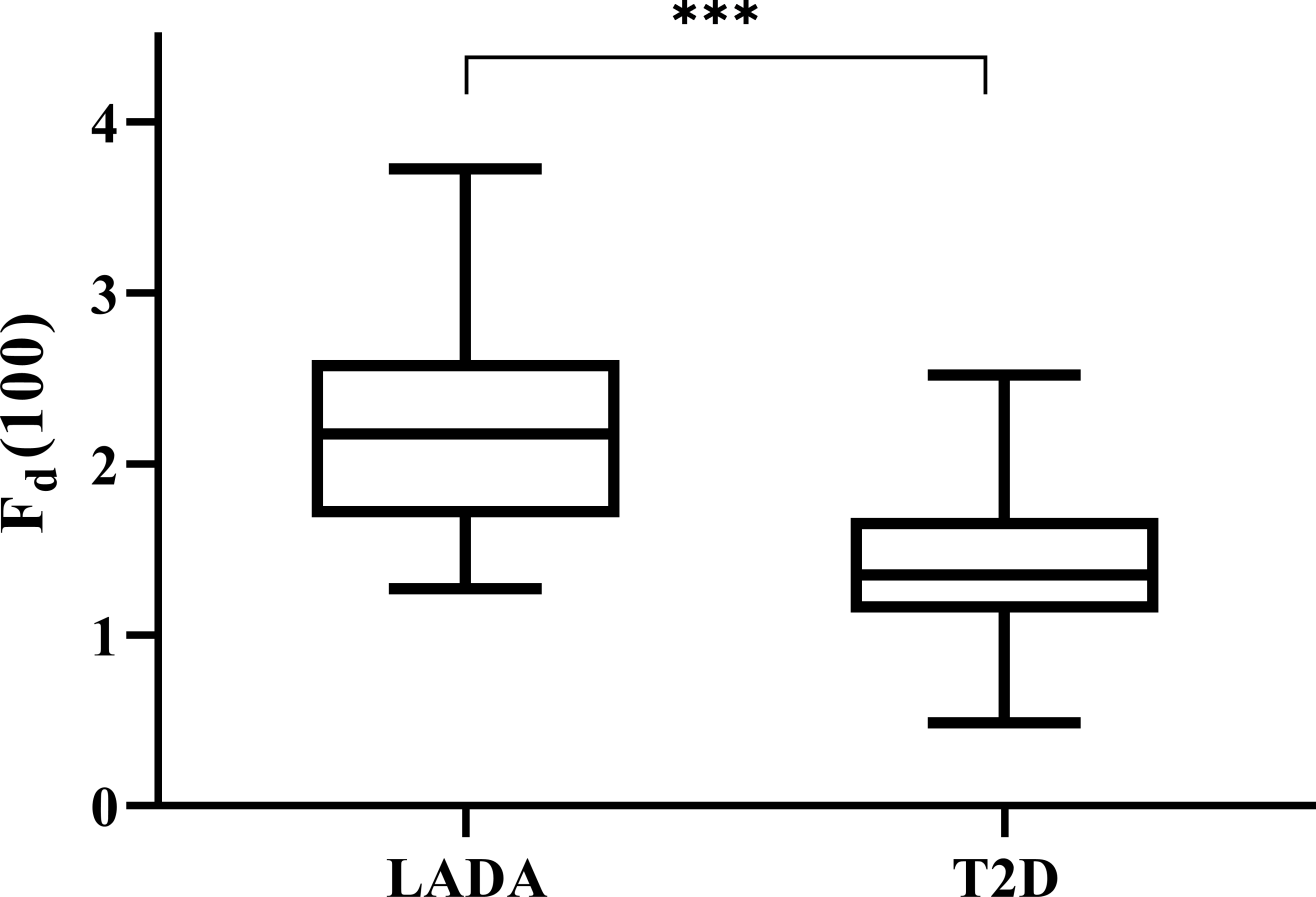
Levels of Fd(100) expressed with box plots of LADA and T2D patients. The top and bottom of the boxes denote the 25th and 75th percentiles, and the line represents the median, the upper and lower endpoint represents the maximum and minimum level respectively. LADA, latent autoimmune diabetes in adults; T2D, type 2 diabetes. ***P < 0.001.

We calculated all the *F_d_(l)* values from *l*=2 to *l*=130 using the glucose data derived from CGM of each patient, and then utilized Spearman correlation analysis to determine the correlation between the corresponding *F_d_(l)* values and beta-cell function parameters (FCP and 2hCP). Supported by the results we got in **Figure 1**, we decided to explore the value of *F_d_(l)* in diabetes differentiation by adopting the scale with the largest correlation coefficient (r=-0.705), that is, *l*=100. The average *F_d_(l)* level of all patients was 1.52 (1.25, 1.98), moreover, of LADA patients was 2.17 (1.67, 2.61) and of T2DM patients was 1.36 (1.14, 1.69), respectively, as displayed in **Figure 2**.

### Spearman correlation analysis

As compared with the classical glucose parameters such as TIR, mean glucose, glucose variability indices including SD of glucose, MAGE and CV, *F_d_(100)* exhibiting a higher correlation coefficient with FCP. TIR, an emerging comprehensive indicator of overall glucose control evaluation, was positively correlated with FCP (r = 0.485, *P*<0.001) and 2hCP (r = 0.548, *P*<0.001). Mean glucose, another commonly used index in clinical practice, was inversely associated with FCP (r = -0.337, *P*<0.001) and 2hCP (r = -0.402, *P*<0.001). For glucose variability parameters, MAGE showed a high correlation coefficient with FCP (r = -0.663, *P*<0.001), and SD showed a strong negative association with 2hCP (r = -0.675, *P*<0.001). However, the *F_d_(100)* displayed the strongest correlation with FCP (r = -0.705, *P*<0.001). Details are shown in **Table 2**.

**Table 2:** Correlation between glycemic parameters and C-peptide levels.

	All participants (*n* = 223)					
	TIR	Mean glucose	SD	MAGE	CV	*F_d_(100)*
FCP	0.485***	-0.337***	-0.645***	-0.663***	-0.639***	-0.705***
2hCP	0.548***	-0.402***	-0.675***	-0.600***	-0.630***	-0.612***

Values represent Spearman correlation coefficients.

FCP, fasting C-peptide; 2hCP, 2-hour postprandial C-peptide; TIR, time in range; SD, standard deviation of glucose; MAGE, mean amplitude of glucose excursions; CV, coefficient of variation. ***P < 0.001.

### ROC analysis and the kappa test in all participants

**Figure 3:**
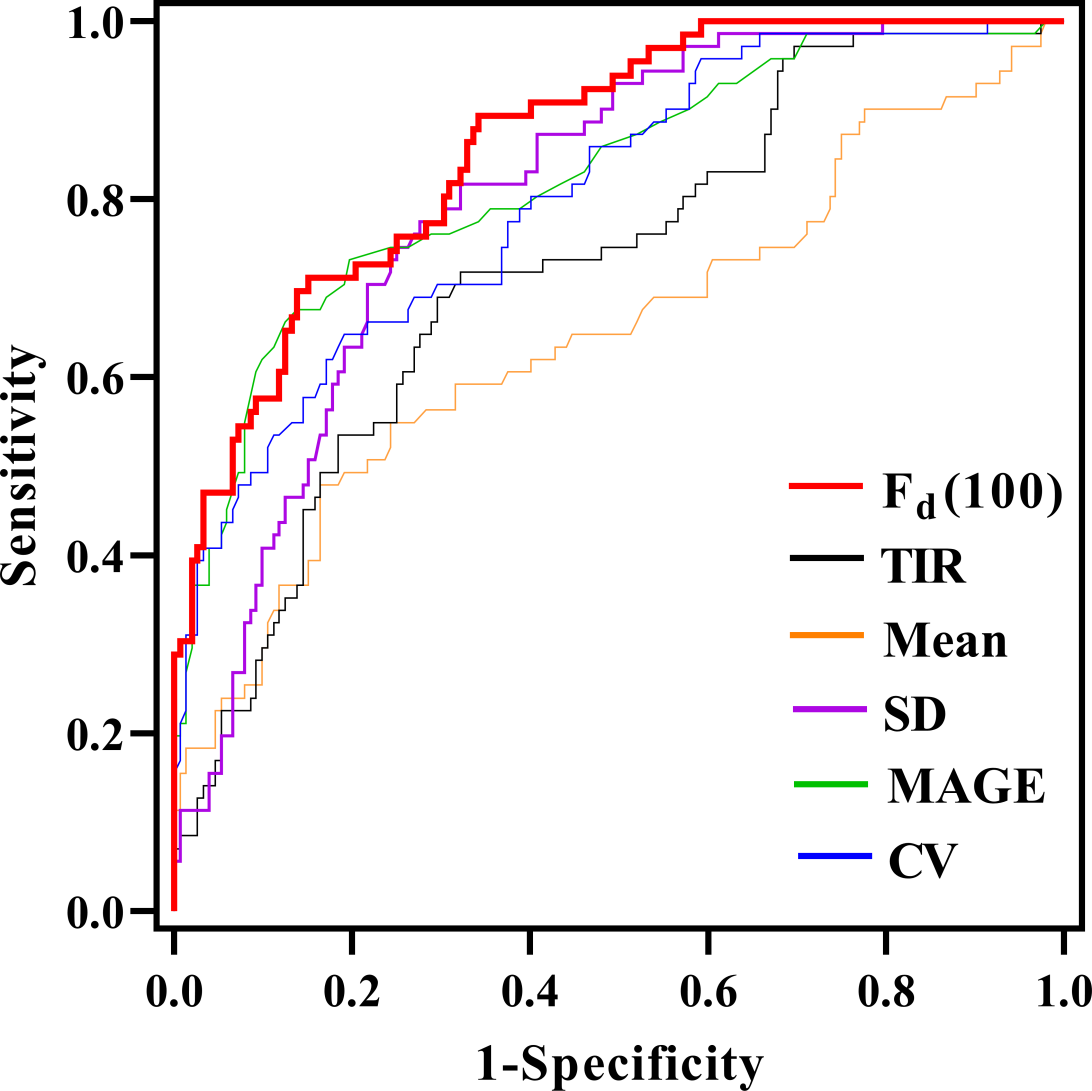
Comparison of the area under the curve (AUC) for glycemic parameters in discriminating between LADA and T2D through receiver operating characteristic curves.

ROC analysis was used to compare the classification performance of *F_d_(100)* and other glycemic parameters, as shown in **Figure 3**. It could be seen that the *F_d_(100)* showed the best performance in differentiating LADA and T2DM patients, the cut-off value was 1.82, achieving an area under curve (AUC) of 0.862 (95% CI [0.813, 0.912], sensitivity: 71.2%, specificity: 84.9%). When the SD was used, the AUC was 0.800 (95% CI [0.737, 0.863], sensitivity: 63.6%, specificity: 80.9%); the AUC of mean glucose, MAGE, CV and TIR were 0.650, 0.820, 0.807 and 0.722 (95% CI [0.567, 0.733], [0.757, 0.883], [0.749, 0.865] and [0.651, 0.793], sensitivity: 47.9%, 72.7%, 78.8% and 71.8%, specificity: 83.6%, 80.3%, 72.4% and 67.8%), respectively.

Furthermore, the kappa test was performed to evaluate the consistency with the real classification given by endocrinologists. *F_d_(100)* presented a good consistency with the real diagnosis (kappa = 0.551, *P*<0.001).

### Ten-fold cross-validation

Ten-fold cross-validation was employed to validate the stability of DFF in the diabetes classification. First, 223 patients were randomly divided into 10 groups. Next, the group 1 to 9 were regarded as the training set, and the group 10 was the test set. Then, the group 1 to 8 and group 10 were the training set, and the group 9 was the test set; repeated 10 times. Results were listed in **Table 3**. The mean AUC of the 10 training sets was 0.863 (95% CI [0.859, 0.868], sensitivity: 78.8%, specificity: 77.8%), and average AUC was 0.866 (95% CI [0.830, 0.903], sensitivity: 80.9%, specificity: 84.1%) in the 10 test sets.

**Table 3:** Crossover-validation of performance of *F_d_(l)* in classification.

Groups	*l*	Training sets			Test sets		
		AUC	Sensitivity (%)	Specificity (%)	AUC	Sensitivity (%)	Specificity (%)
1	101	0.866	71.2	85.3	0.821	71.4	87.5
2	101	0.853	89.5	65.9	0.929	88.9	92.9
3	97	0.865	72.9	85.6	0.901	85.7	92.3
4	101	0.872	90.0	66.7	0.777	72.7	81.8
5	101	0.867	73.0	85.0	0.816	75.0	68.4
6	107	0.871	70.5	87.6	0.844	83.3	80.0
7	102	0.858	88.3	67.2	0.922	83.3	86.7
8	102	0.858	88.3	64.7	0.902	83.3	88.2
9	101	0.867	73.3	84.6	0.857	85.7	68.7
10	98	0.860	70.5	85.2	0.894	80.0	94.1
Mean	101.1	0.863	78.8	77.8	0.866	80.9	84.1
95% CI	99.2, 103.0	0.859, 0.868	72.4, 85.1	70.6, 85.0	0.830, 0.903	76.7, 85.2	77.4, 90.7

AUC, area under curve; CI, confidence interval.

### ROC analysis and the kappa test in the insulin-treated population

One-hundred and sixteen participants treated with insulin (66 LADA and 50 T2DM, **Table 4**) were further included in the ROC analysis to validate the performance of DFF. *F_d_(100)* yielded an AUC of 0.842 (95% CI [0.771, 0.913], sensitivity: 72.1%, specificity: 84.0%), the cut-off value of *F_d_(100)* in this population was 1.84 (data not shown). Moreover, *F_d_(100)* also presented a satisfactory consistency with the real diagnosis (kappa = 0.552, *P*<0.001).

**Table 4:** Insulin use of all participants.

	LADA (n = 71)	T2DM (n = 152)
Type of insulin treatment, n (%)		
MDI	45 (63.4)	4 (2.6)
Basal insulin dose (U/kg·d)	0.1882	0.2915
glargine/degludec/detemir/NPH	33/10/0/2	3/1/0/0
Bolus insulin dose (U/kg·d)	0.2886	0.3272
CSII	4 (5.6)	0
Basal rate (U/kg·d)	0.3732	/
aspart/lispro	2/2	/
Bolus insulin dose (U/kg·d)	0.2316	/
Only basal insulin regimen	10 (14.1)	13 (8.6)
Insulin dose (U/kg·d)	0.1850	0.2257
glargine/degludec/detemir/NPH	8/2/0/0	8/3/1/1
Only premixed insulin regimen	7 (9.8)	33 (21.7)
Insulin dose (U/kg·d)	0.4738	0.4284

MDI, multiple daily injections; NPH, neutral protamine hagedorn; CSII, continuous subcutaneous insulin infusion.

## Discussion

The term LADA is acceptable in clinical practice for its practical impact of highlighting proper treatment and insulin initiation prior to beta-cell function failure (16–18). Jones et al. (19) suggested that LADA may represent a mixed population of autoimmune diabetes (type 1) and non-autoimmune diabetes (type 2). Although the quality of modern islet autoantibody detection has improved (20), abnormally high specificity is required in low-risk groups with rare GADA antibody positivity such as patients with T2DM. The LADA International Expert Panel recommended to measure serum C-peptide levels as a proxy of insulin secretion in patients with positive islet cell-associated autoantibodies (21, 22), since the decline rate of C-peptide in LADA is midway between T1DM and T2DM (5, 23, 24).

The present study was based on the remarkably different beta-cell function in patients with LADA and T2DM, thus resulting in different pattern in glucose variability and other glycemic indices. We investigated the value of calculated DFF based on numerous glucose data retrieved from CGM, and compared the performance of DFF with several classical glucose parameters in distinguishing LADA and T2DM patients. As we know, studies reporting glucose fluctuation patterns in patients with LADA are scarce, and we did notice that glucose variability in LADA was significantly greater than that of T2DM patients who were matched for age and diabetes duration. Consequently, based on this result, we found that the correlation between DFF and beta-cell function assessed by FCP was strongest using *F_d_(100)* values obtained at an appropriate time scale of *l* = 100 (when the time period of the segmented glucose sequence was 8 hours and 15 minutes). Moreover, we further explored the performance of *F_d_(100)* in diabetes classification with ROC analysis, and we noted that *F_d_(100)* yielded a remarkable value as compared with the current commonly used glucose parameters.

The DFF calculation method we adopted was derived from detrended fluctuation analysis (DFA), a parameter evaluating glucose complexity. In general, it is considered to represent the long-term temporal auto-correlation rather than the glucose variability (13). DFA is supposed to mirror the intrinsic properties of individuals with different glucose metabolism status whether in normal subjects, prediabetic or diabetic patients. A study indicated that higher DFA was associated with worse glucose control in patients with diabetes (25). In addition, DFA was shown to be able to estimate insulin resistance either in healthy individuals or in T1DM (26), predict the probability of developing T2DM in patients at risk (27), and assess mortality in ICU patients (28). In our preliminary analysis, we found a significant difference in DFA levels between patients with LADA and T2DM, but the performance of DFA in diabetes classification was not satisfactory. Inspired by Liu et al. (14), we explored the value of DFF in distinguishing patients with LADA and T2DM. DFF was generated when the optimal time scale was selected on the basis of the calculation of DFA, in order to maximize the ability in reflecting the endogenous insulin secretion in our study population. As *l* increased, the correlation between *F_d_(l)* and FCP, 2hCP increased gradually and tended to be stable. Eventually, *F_d_(100)* was selected based on the characteristics of the glucose sequence generated by the CGM system we used. Several previous studies had reported that classical CGM-derived glycemic parameters such as MAGE and CV were closely related to beta-cell function (29–31). In our study, *F_d_(100)* showed a stronger correlation with FCP compared with TIR, mean glucose, SD, MAGE and CV. Similarly, levels of *F_d_(100)* were significantly higher in patients with LADA than that in patients with T2DM. ROC curves were employed to test the performance of *F_d_(l)* and classical glycemic parameters in distinguishing patients with LADA from T2DM. As expected, *F_d_(l)* yielded the largest AUC and achieved a high specificity (84.9%). Furthermore, the performance of *F_d_(l)* was verified to be stable in 10 groups of training and test sets with a 10-fold cross-validation method.

Emerging evidence suggests that CGM provides important information about glycemic variability that have direct implications for the glucose regulation of patients with diabetes. Several studies have indicated the potential role of CGM data in distinguishing people with different glucose metabolism. For example, CGM measures of hyperglycemia and glycemic variability were validated to be superior to HbA1c in distinguishing those with and without cystic fibrosis related diabetes (CFRD), indicating CGM as a diagnostic and screening tool for CFRD (32). Another Two studies developed a polynomial-kernel support vector machine-based approach and demonstrated the ability to distinguish between subjects affected by impaired glucose tolerance (IGT) and T2DM based on a pool of glycemic variability indices complemented by four basic parameters-age, sex, BMI, and waist circumference (33, 34). Hall et al. (35) introduced the concept of “glucotypes” that has attracted enormous attention in precision medicine. They developed an algorithm to identify patterns of glycemic variability based on CGM and argued that glucotypes provide the advantage of taking into account a more detailed picture of glucose dynamics compared with commonly used average-based measures, revealing subphenotypes within traditional diagnostic categories of glucose regulation. We found that CGM data derived measure-DFF significantly correlated with C-P, and that these correlations were stronger than commonly used CGM glycemic variability indices. These findings suggest that the information obtained by DFF is clinically meaningful and perhaps more relevant for clinical care than SD, MAGE or CV in diabetes.

Glucose-lowering medication in our LADA and T2DM patients was different. Approximately ninety percent of our LADA patients were treated with insulin, which is larger than that of T2DM patients. Apparently, insulin treatment is bound to affect both C-P secretion and CGM-related results. Herein, we included all insulin-treated LADA and T2DM participants in additional ROC analysis and kappa test to test the stability of DFF. And supported by an AUC of 0.842 (sensitivity: 72.1%, specificity: 84.0%) and a kappa value of 0.552, the added value of DFF in diabetes classification was further validated in identifying both insulin-dependent LADA and T2DM patients in our study population. However, given that LADA patients in our study population were almost insulin treated, we were not able to evaluate the potential value of DFF in identifying insulin-naïve LADA patients, which may be a limitation of our study.

As previously mentioned, the GADA testing is the recommended screening tests for LADA because of its known prediction of β-cell function decline in patients with LADA (4), and screening autoimmune diabetic patients among T2DM patients requires extremely higher specificity (19). Therefore, even if computed specificity in our study was inferior to that of GADA detection in clinical practice, phenotypic T2DM patients could be suspected as LADA by *F_d_(l)* calculation, which might improve the diagnostic rate of LADA patients. Ultimately, large long-term prospective studies will be needed to investigate if DFF will similarly predict β-cell function decline in LADA. In the meantime, identifying CGM measures that correlate with the C-P levels of LADA and T2DM patients establishes an important first step in this process, particularly given the notable benefits of using CGM-derived DFF in this setting. Apparently, obtaining CGM data by simply placing a sensor at a clinic visit is easy and convenient, offering the potential to substantially improve LADA screening rates. In addition, CGM wearing would provide a comprehensive assessment of glucose control, allowing for the identification of glycemic patterns to guide individualized management decisions and insulin therapy in an efficient manner.

Unsurprisingly, our results were not as good as those obtained by adopting *F_d_(l)* to distinguish T1DM from T2DM (14). Since T1DM is known as ‘fragile diabetes’, absolute insulin deficiency and lifelong insulin-dependent treatment render great glucose fluctuations and frequent hypoglycemia in this population (36). Nevertheless, patients with T2DM who are insulin resistant always present mainly hyperglycemia and rare hypoglycemia, consequently undergo a much smaller glucose variability than that of T1DM. Theoretically, the application of *F_d_(l)* in differentiating T1DM from T2DM would be more effective. Moreover, the insulin secretory capacity of our LADA patients was nearly three times as their T1DM patients, we here broaden the application of DFF in diabetes classification. Last but not least, differentiating LADA from T1DM is surely an important step to validate the clinical significance of DFF since LADA is almost T1DM-phenotypic as the diabetes progresses. LADA shares the autoimmune pathogenesis of T1DM, except that the immune damage to pancreatic β-cells of LADA progresses slower than that of T1DM. Some LADA patients with diabetic ketoacidosis (DKA) onset are likely to be misdiagnosed as T1DM. Moreover, in patients with T1DM at a stage of partial recovery of islet function, such as the honeymoon stage, their insulin secretory capacity may be close to that of LADA patients. With gradual adoption of CGM, it would be of great interest to fully understand the information carried by the numerous glucose data and consequently apply to the precision medicine of diabetes.

To the best of our knowledge, this is the first study to distinguish patients with LADA and T2DM by using CGM data derived parameters. Consistent with the reported studies (8), we marked a more violent glucose fluctuation pattern in patients with LADA. At present, early diagnosis of LADA patients remains a challenge in China. The LADA international Expert Panel recommended that all newly diagnosed T2DM patients should be screened for GADA positivity and follow-up of progressing beta-cell failure, which might increase the burden of medical care (21). Undoubtedly, GADA positivity, C-P levels and slim body are valuable for differential diagnosis of LADA or T2DM. However, we here provided an additional proof for diabetes classification by calculating *F_d_(l)* as the CGM system is increasingly widely used in glucose management.

We acknowledge that there were a few limitations. First, the sample size of patients with LADA was relatively small compared with that of T2DM, potentially limiting the statistical power in this group of individuals. Second, our findings of cut-off thresholds need to be validated in patients immediately after diagnosis of LADA since insulin treatment is bound to affect both C-P and glucose parameters. There were 2 patients taking pioglitazone in the T2DM group, and we did not evaluate their tiny effect on C-peptide release and blood glucose levels. Third, potential biases caused by uncertain confounding factors in such a cross-sectional and single-center study were difficult to rule out completely. In order to improve the clinical significance of DFF in this study, we will further explore the performance of DFF in other newly-diagnosed, untreated LADA and T2DM patients in the future. Moreover, various patients with specific diabetes diagnosis will also be collected to further validate our data-driven analysis.

To summarize, DFF was able to identify nearly 80 percent of patients with LADA from T2DM in our study population, which may provide additional proof for diabetes classification. At the same time, our study broadened the application of data processing method in the field of diabetes classification. Larger sample size and multi-center research would be focused on the validation and optimization of this data processing method in the future, aiming to make a great effort for precision medicine in diabetes.

## Data availability statement

The raw data supporting the conclusions of this article will be made available by the authors, without undue reservation.

## Ethics statement

The studies involving human participants were reviewed and approved by The Ethics Review Committee of the Second Xiangya Hospital of Central South University. The patients/participants provided their written informed consent to participate in this study.

## Author contributions

LY and ZZ designed the study and revised the manuscript, LZ and QT conducted the data analysis and wrote the draft of the paper. KG, JW, JY, ZD, QZ helped to prepare and collect the data. GH performed the GADA assay. Thanks to XL who kindly offered administrative support in data collecting. The corresponding author attests that all listed authors meet authorship criteria. All authors contributed to the article and approved the submitted version.

## Funding

This work was supported by the National Key R&D Program of China (2018YFC2001005).

## Acknowledgments

The authors express special gratitude to all the patients and staff who participated in this study. We would like to thank associate researcher Dr. Jingzhen Li from Shenzhen Institute of Advanced Science and Technology for assisting us in data processing.

## Conflict of interest

The authors declare that the research was conducted in the absence of any commercial or financial relationships that could be construed as a potential conflict of interest.

## Publisher’s note

All claims expressed in this article are solely those of the authors and do not necessarily represent those of their affiliated organizations, or those of the publisher, the editors and the reviewers. Any product that may be evaluated in this article, or claim that may be made by its manufacturer, is not guaranteed or endorsed by the publisher.
